# Evaluating automatic annotation of lexicon-based models for stance detection of M-pox tweets from May 1st to Sep 5th, 2022

**DOI:** 10.1371/journal.pdig.0000545

**Published:** 2024-07-30

**Authors:** Nicholas Perikli, Srimoy Bhattacharya, Blessing Ogbuokiri, Zahra Movahedi Nia, Benjamin Lieberman, Nidhi Tripathi, Salah-Eddine Dahbi, Finn Stevenson, Nicola Bragazzi, Jude Kong, Bruce Mellado

**Affiliations:** 1 School of Physics and Institute for Collider Particle Physics, University of the Witwatersrand, Johannesburg, South Africa; 2 iThemba LABS, National Research Foundation, Cape Town, South Africa; 3 Africa-Canada Artificial Intelligence and Data Innovation Consortium (ACADIC), York University, Toronto, Canada; 4 Laboratory for Industrial and Applied Mathematics, York University, Toronto, Canada; 5 Department of Computer Science, Brock University, St. Catharines, Niagara Region, Ontorio, Canada; 6 Department of Mathematics, Bahen Center for Information Technology, University of Toronto, Canada; 7 Global South Artificial Intelligence for Pandemic Preparedness and Response Network (AI4PEP), York University, Toronto, Canada; 8 Artificial Intelligence & Mathematical Modeling Lab (AIMMLab), Dala Lana School of Public Health, University of Toronto, Canada; Dalhousie University Faculty of Computer Science, CANADA

## Abstract

Manually labeling data for supervised learning is time and energy consuming; therefore, lexicon-based models such as VADER and TextBlob are used to automatically label data. However, it is argued that automated labels do not have the accuracy required for training an efficient model. Although automated labeling is frequently used for stance detection, automated stance labels have not been properly evaluated, in the previous works. In this work, to assess the accuracy of VADER and TextBlob automated labels for stance analysis, we first manually label a Twitter, now X, dataset related to M-pox stance detection. We then fine-tune different transformer-based models on the hand-labeled M-pox dataset, and compare their accuracy before and after fine-tuning, with the accuracy of automated labeled data. Our results indicated that the fine-tuned models surpassed the accuracy of VADER and TextBlob automated labels by up to 38% and 72.5%, respectively. Topic modeling further shows that fine-tuning diminished the scope of misclassified tweets to specific sub-topics. We conclude that fine-tuning transformer models on hand-labeled data for stance detection, elevates the accuracy to a superior level that is significantly higher than automated stance detection labels. This study verifies that automated stance detection labels are not reliable for sensitive use-cases such as health-related purposes. Manually labeled data is more convenient for developing Natural Language Processing (NLP) models that study and analyze mass opinions and conversations on social media platforms, during crises such as pandemics and epidemics.

## Author summary

Social media platforms are pivotal in shaping public opinion during health crises, influencing policy-making and crisis management. Challenges such as labor-intensive manual labeling and dataset biases highlight the need for optimized stance detection methods. Our study assessed VADER and TextBlob for stance detection during the M-pox outbreak on social media, comparing their automated labels with our manually labeled data. Transformer-based models consistently outperformed lexicon-based approaches, showing significant improvements both before and after fine-tuning. Specifically, models pre-trained on the COVID-19 tweets demonstrated over a 20% enhancement in accurately classifying M-pox tweets. Through topic modeling of misclassified tweets, nuanced sub-topics in M-pox discussions were identified, highlighting the value of integrating multi-modal data and using hand-labeled datasets for comprehensive sentiment analysis across platforms and contexts. Policymakers and healthcare authorities can utilize these insights to craft precise communication strategies, combat misinformation, and address public concerns effectively. Advancements in machine learning for health-related stance detection hold promise for optimizing crisis management and informing evidence-based policy-making during emerging epidemics and pandemics, with implications for future research and policy development.

## Introduction

Stance detection plays a crucial role in understanding the opinions and attitudes expressed in text towards a specific target. Unlike sentiment analysis, which focuses on determining the emotional tone of a text, stance detection aims to identify whether the author is in favor of, against, or neutral towards the target. This distinction is particularly important in the context of social media, where individuals express diverse and sometimes conflicting viewpoints [1].

While both stance detection and sentiment analysis have been extensively used in analyzing social media posts, there is a need to evaluate the performance of automated labeling approaches, especially in the domain of stance detection [2, 3]. Traditionally, sentiment analysis and stance detection models were developed using hand-labeled data, which is labor-intensive and time-consuming. In recent years, there has been a shift towards using lexicon-based models for automatic labeling, such as VADER and TextBlob [3]. These models assign positive, neutral, or negative polarities to words in a sentence, enabling automated sentiment or stance classification [4–6].

However, few studies have focused on evaluating the performance of lexicon-based models specifically for stance detection [7]. In this study, we aim to fill this gap by comparing the accuracy of automated labeled data using VADER [8] and TextBlob [9] against models fine-tuned on a manually labeled dataset related to M(onkey)-pox tweets. We focus on tweets related to the M-pox outbreak from May 1st to Sep 5th, 2022, with a particular emphasis on the government’s response to the outbreak.

Specifically, we aim to answer three questions in this study:

- How could we evaluate automated labeling for stance detection?
- How could we compare the performance of transformer-based models against automated labels?
- How does fine-tuning on transformer-based models change the scope of the misclassified tweets?

To achieve this, we manually label a Twitter dataset related to M-pox based on insights into the government’s response to the outbreak. We then compare the accuracy of automatic labeled data using lexicon-based models against models fine-tuned on the hand-labeled M-pox dataset for stance detection. Our study differs from previous works by fine-tuning four different transformer-based models on manually labeled data and comparing their accuracy against lexicon-based automated labels.

The significance of this study lies in its potential to improve the accuracy of stance detection models in sensitive domains such as healthcare. By providing a meticulously hand-labeled dataset related to the government’s response to M-pox, we aim to contribute to the development of more accurate stance detection models that can be used for analyzing public opinion during health crises.

The remainder of this paper is structured as follows: section two reviews related work on lexicon-based models for text labeling and sentiment analysis of M-pox tweets. Section three describes the methodology, including data collection, preparation, and analysis. Section four presents the findings of our study. In section five, we discuss the implications of our results and compare them with related work. Finally, section six summarizes the key findings of the study and outlines directions for future research and policy implications.

## Related works

During previous pandemics, social and healthcare workers have always struggled with issues such as stigmatization, vaccine hesitancy, rumors and fake news dissemination, mis- and dis-information, conspiracy theories, and disagreements with pharmaceutical Interventions (PI) and Non-Pharmaceutical Interventions (NPI) [10]. In recent pandemics such as COVID-19 and M-pox, this has been exacerbated by social media influencers [11, 12]. Therefore, different parties including researchers, decision makers, and health officials have urged to develop NLP models to study and analyze discussions and conversations on social media platforms. Studying discussions and mass opinions on social media enable informed policies that mitigate these issues and bring back public trust and cooperation [13–15]. For instance, Alotaibi, et al [16] applied topic modeling on tweets to understands causes of vaccine rejection during COVID-19 pandemic. Khan, et al [17] used Twitter to identify individual and community factors that cause vaccine hesitancy. Ogbuokiri B, et al [18] used Twitter to find vaccine hesitancy hotspots in South Africa on city-level, and in [19] they tried to understand the post-vaccination sentiment in Africa. This work verifies the superiority of hand-labeled data to automated labeled data by comparing lexicon-based automated labels with different transformer-based models; therefore, it could help developers build more efficient and dependable NLP models for disease control and emergency management in future outbreaks and epidemics [11–13, 20].

Stance detection and sentiment analysis have been extensively studied in the context of social media analysis. Previous research has primarily focused on evaluating automated labeling for sentiment analysis [21–23]. Although lexicon-based models are commonly used for stance detection automatic labeling [3, 5, 6], few studies have specifically evaluated the performance of lexicon-based models for stance analysis [24].

Recent advancements in NLP have introduced transformer-based models, which have shown promising results in various NLP tasks, including sentiment and stance analysis [25–27]. These models have not only improved performance but also provided a new perspective for analyzing text data. In 2017, Google brain released the first transformer-based model to revolutionize the world of NLP [25]. Since then, numerous models have been fine-tuned on transformer pre-trained models for sentiment and stance analysis [26, 27]. However, previous works have not compared the performance of lexicon-based automated labels against transformer based models.

Our work is different from previous works in the sense that we fine-tune four different transformer models on manually labeled data and compare their accuracy against lexicon-based, i.e. VADER and TextBlob, automated labeled data. Two of the transformer-based models include NLP-Town BERT [28] and Cardiff-NLP RoBERTa [29], and the other two are borrowed from [4], i.e. COVID-19 BERT and COVID-19 RoBERTa models. In [4], BERT [30] and RoBERTa [31] were fine-tuned for stance detection of tweets related to COVID-19 vaccines. Although these models were trained on a different dataset (COVID-19 vaccine-related tweets), they demonstrated higher accuracy compared to TextBlob automated labels. This suggests that training models on a closely related but different dataset can improve accuracy beyond lexicon-based automated labeling.

Building on this, our work aims to evaluate lexicon-based models for stance detection of M-pox tweets. We manually labeled a Twitter dataset related to M-pox based on insights into the government’s response to the 2022 M-pox outbreak. By comparing the accuracy of automatic labels using VADER and TextBlob against models fine-tuned on our M-pox dataset, we aim to assess the effectiveness of lexicon-based models in this context. Furthermore, we fine-tune four transformer-based pre-trained models (COVID-19 BERT, COVID-19 RoBERTa, NLP-Town BERT, and Cardiff-NLP RoBERTa) on our manually labeled M-pox dataset. Our results show that these transformer-based models outperform VADER and TextBlob automated labels, indicating the potential of transformer models in stance detection of M-pox tweets.

## Materials and methods

### Sample and data

Out of approximately 100,000 tweets that were collected using the Twitter Research License, a total of 20604 tweets were selected using the cluster sampling method proposed in [4] for hand-labeling. The extraction focused on hashtags relating to M-pox over a time period spanning from the 1^*st*^ May 2022 to the 5^*th*^ September 2022—which is the period before and after the peak in the global infections [32]. The same hand-labeling rules and procedures performed in [4] were used for labeling this M-pox dataset which included 22.2% positive, 35.3% neutral, and 42.5% negative tweets. Our manually labeled M-pox dataset is available as a supplementary csv file to this manuscript (S1). Due to Twitter’s developers’ policy [33], only Tweet IDs can be shared with the public. Therefore, this dataset (S1) includes only two columns, namely, TweetID and Label, which present the ID of the tweet, and its stance label, respectively. To retrieve the actual text of the tweet and other metadata such as creation date, the tweet IDs need to be hydrated [34]. A label is ascribed to the tweet based on the answer to the opinion of the author towards the following question:

- A negative sentiment was defined as an overwhelming and irrational feeling of fear and impending doom accompanied by distrust or poor faith in the government’s ability to control the M-pox Outbreak.
- A positive sentiment was defined as the absence of unwarranted fear accompanied by a strong belief and deep trust in the government.
- A neutral sentiment was defined as the refusal to engage in discussions pertaining to the threat of M-pox as a public health hazard and the possible threat of a new pandemic, due to indifference, disinterest or an indecisive temperament towards the severity and planned mitigation of the M-pox Outbreak.

### Measures of variables

After labeling the dataset, it was used to further fine-tune the pre-trained models, COVID-19 BERT and COVID-19 RoBERTa models presented in [4], as well as NLP-Town BERT [28] and Cardiff-NLP RoBERTa [29]. For BERT and RoBERTa, the same values found in [4] for hyperparameters, i.e. learning rate, weight decay = 0, batch size, and number of epochs, were accepted and used in this work. Moreover, for NLP-Town BERT and Cardiff-NLP RoBERT, the learning rate was set at 0.0001 and 0.001 (weight decay = 0 for both), the batch-size was equal to 32 and 64, and the number of epochs agreed with 8 and 10, respectively. Unlike [4], no pre-processing was performed prior to training, as all the models have their own built-in Tokenization schemes unique to the model. The COVID-19 RoBERTa model provided the highest accuracy among all the fine-tuned models. Therefore, topic modeling was performed on the misclassifications of the pre- and post-fined-tuned COVID-19 RoBERTa models. Maximum coherence for both of the models was obtained when the number of topics was equal to 5.

### Models and data analysis procedure

One-fifth of the Mpox dataset was selected as the testing dataset (2843 tweets), and the rest as the training dataset. Two well-known models, namely, VADER [8] and TextBlob [9] were used for automated labeling of the M-pox testing dataset. The labels were then compared against the hand-labels to examine the accuracy. Mislabeled data were grouped into four categories:

- Clear-cut cases correspond to tweets whose stance labels are obvious, and there is no debate on the validity of its classification—in other words, the tweet’s polarity is heavily skewed towards a single stance type.
- Borderline cases correspond to tweets that can arguably take on one of two labels i.e., neutral or positive or alternatively neutral or negative, whereby the author’s point of view is debatable.
- Difficult-to-label tweets are tweets that contain both positive and negative stances, each with high polarity scores, which makes it difficult to decide on the overall text polarity.
- Same-text tweets have the same raw text but differ in the amount of punctuation and/or emojis present in the tweet, which serves to change the message behind the tweet, often through satire.

**Fig 1:**
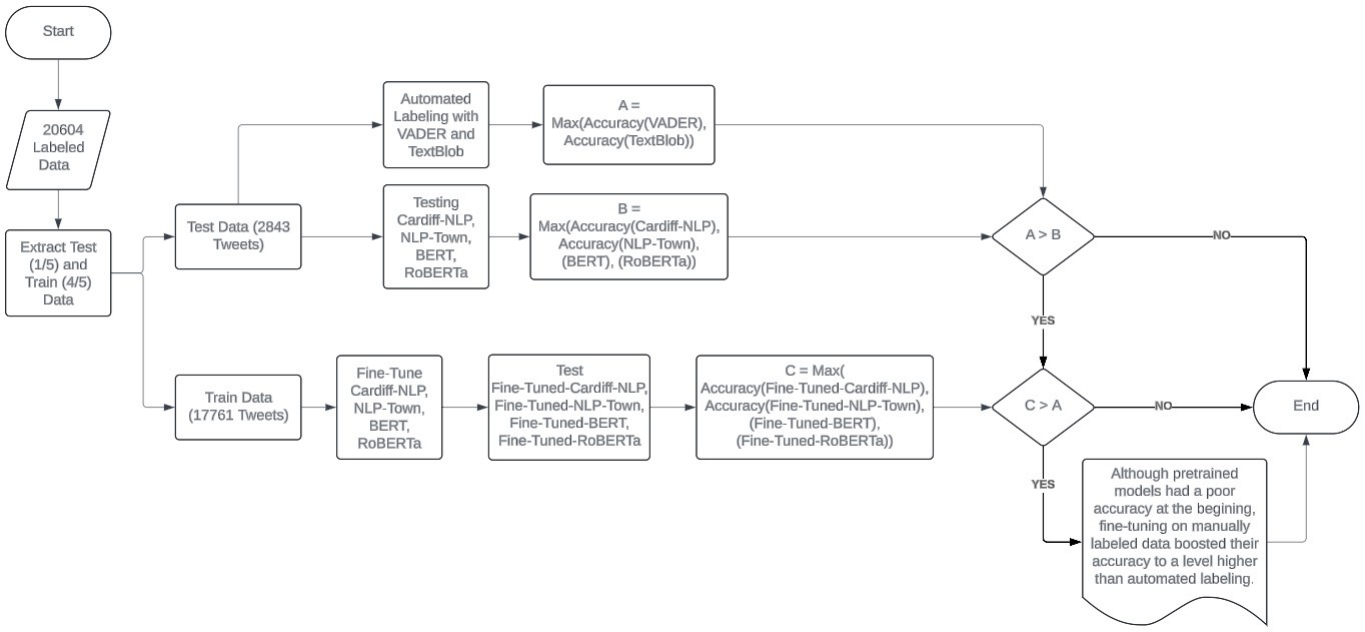
The logic of the study.

Next, the COVID-19 BERT and COVID-19 RoBERTa models presented in [4], as well as two other pre-trained models, i.e., NLP-Town BERT [28], Cardiff-NLP RoBERTa [29], were tested on the M-pox testing dataset, then fine-tuned on the M-pox training dataset and tested again on the M-pox testing dataset. The testing results before and after fine-tuning were compared with the VADER and TextBlob labeling results, to verify the superiority of hand-labeling against automated labeling. Although the maximum accuracy of the automated labels, which belonged to VADER, was higher than the accuracy of all of the four pre-trained models before fine-tuning, the accuracy of all of the fine-tuned models were higher than VADER after fine-tuning. This shows that automated labeling provides a significantly lower performance compared to fine-tuning on hand-labeled data. Fig 1 shows the flowchart of our study.

To further study how fine-tuning on hand-labelled data has changed the model predictions a Latent Dirichlet Allocation (LDA) was performed, on the tweets misclassified by the RoBERTa model before fine-tuning and after that, since the RoBERTa model provided the highest accuracy among all the four pre-trained models, before and after fine-tuning. The aim of this was to determine which of the topics present in the misclassifications of the initial COVID-19 RoBERTa model remained, disappeared, or emerged after fine-tuning. The top 30 most salient terms in each topic were extracted and the topics were visualized using the pyLDAvis tool and bar charts.

## Results

### Machine learning models

Table 1 presents the accuracy of data labeled by VADER and TextBlob against hand-labeled data. The first column of Table 1 represents the performance indicator (Precision, Recall, F-score, and Accuracy), and for each model, the first three columns are associated to the three classes, i.e. Negative, Neutral, Positive, and the fourth column represents overall model performance.

**Table 1:** Accuracy of automated labeling using VADER and TextBlob. The M-pox dataset is from May 1st to Sep 5th, 2022.

	VADER Algorithm				TextBlob Algorithm			
Class	Negative	Neutral	Positive	All	Negative	Neutral	Positive	All
Precision	45	52	50	49	35	40	47	41
Recall	47	71	20	50	37	62	16	40
F1-score	46	60	28	47	36	49	23	37
Accuracy				50				40

Next, mislabeled data were grouped into four distinct categories, namely, clear-cut, border-line, difficult, and same text. Table 2 presents an example of each category. In Table 2, “+”, “0”, and “-” represent positive, neutral, and negative stances, respectively. Emojis are represented by their descriptions as written text in brackets.

**Table 2:** An illustration of the advantages of Manual over Automated text labeling. The M-pox dataset is from May 1st to Sep 5th, 2022.

Cases	Tweet	Hand	VADER	TextBlob
Clear-cut	We beat COVID, now let’s beat M-pox. (flexed bicep) (syringe) #M-pox	+	+	+
Clear-cut	M-pox is a hoax! Get real, people!	-	-	0
Clear-cut	M-pox does not seem to be as deadly as everyone says	0	0	0
Border-line	My family got M-pox. All had minor symptoms and are now 100%.	+	0	0
Border-line	A M-pox Outbreak right after COVID ends? Hmm…(thinking-face)	-	0	0
Border-line	Now there is M-pox. (worried-face) Seriously? (frowning face) Should I be concerned? (thinking face)	0	-	-
Difficult	I’ll take precaution, but if I get it and die, then I die. Oh well!	+	-	0
Difficult	I have fully recovered from M-pox, but a friend got it and died.	-	-	0
Difficult	M-pox can infect people of any race and any sexual orientation.	0	0	0
Same Text	WHO is doing a great job. Let’s support them!! (praying hands)	+	+	0
Same Text	WHO is doing a great job. Let’s support them!! (laughing face)	-	+	0
Same Text	WHO is doing a great job. Let’s support them??	0	+	0

Overall VADER correctly predicted the labels of 50% of the tweets, in which 100% of the clear-cut case examples were classified correctly, while none, or 0%, of the border-line case tweets were classified correctly and only one third, 33%, of the difficult-to-label or the same text tweets were correctly labelled. VADER was able to get 50% recall for each respective class.

Comparatively, TextBlob correctly predicted the labels of a third, or ≈ 33%, of all the tweets, in which two thirds, or ≈ 67%, of the clear-cut case examples were classified correctly, while none, or 0%, of the border-line case tweets were classified correctly, 33% of all of the difficult-to-label tweets were labelled correctly, but none, 0%, of the same text tweets were correctly labelled. TextBlob got recalls of 25% for the positives, 75% for the neutrals but nothing, 0%, for the negatives.

This shows that both classification algorithms perform well on simple clear-cut examples, but become much less efficient in correctly classifying tweets, as the complexity of the tweets increases. Furthermore, given the recall values, it is apparent that VADER is equally good in labeling each stance type, while TextBlob strongly favours a neutral label. In both cases, the overall accuracies are very low in comparison to hand-labeling and it is clear that when given same text tweets, the algorithms are unable to identify sarcasm or the nuanced effect of changing punctuation marks i.e., from ! to ?, given that VADER provided a positive label for each stance belonging to the same text case, while TextBlob provided all neutral labels.

Table 3 evaluates the pre-trained models through four different metrics namely, precision, recall, F1-score, and accuracy, using the M-pox testing dataset, before and after fine-tuning. Similar to Table 1, the first column of Table 3 represents the performance indicator (Precision, Recall, F-score, and Accuracy). For each model, i.e. VADER Algorithm and TextBlob Algorithm, the first three columns are associated to the three classes, i.e. Negative, Neutral, Positive, and the fourth column, i.e. All, represents the overall performance of the model.

**Table 3:** COVID-19 vs pre-trained Model Performances on M-pox data. The M-pox dataset is from May 1st to Sep 5th, 2022.

	NLP-Town BERT				Cardiff-NLP RoBERTa			
Class	Negative	Neutral	Positive	All	Negative	Neutral	Positive	All
Precision	48	40	41	43	46	38	37	40
Recall	37	56	22	39	35	59	26	40
F1-score	42	47	30	40	40	46	31	39
Accuracy				39				40
	COVID-19 BERT				COVID-19 RoBERTa			
Class	Negative	Neutral	Positive	All	Negative	Neutral	Positive	All
Precision	56	43	43	49	56	44	40	48
Recall	42	71	21	47	40	72	21	47
F1-score	48	54	28	45	47	54	28	45
Accuracy				47				47
	Fine-Tuned NLP-Town				Fine-Tuned Cardiff-NLP			
Class	Negative	Neutral	Positive	All	Negative	Neutral	Positive	All
Precision	59	68	57	60	55	67	59	58
Recall	63	60	60	61	67	56	71	62
F1-score	61	63	58	60	60	61	64	60
Accuracy				60				59
	Fine-Tuned BERT				Fine-Tuned RoBERTa			
Class	Negative	Neutral	Positive	All	Negative	Neutral	Positive	All
Precision	69	73	60	69	67	77	64	70
Recall	72	71	57	68	77	68	57	69
F1-score	70	72	58	68	72	72	61	69
Accuracy				68				69

Before fine-tuning, the pre-trained NLP-Town BERT model achieved an overall precision of 43%, an overall accuracy of 39% yielding an F1-score of 40%, while the pre-trained Cardiff-NLP RoBERTa model achieved a similar result with an overall precision of 40%, overall accuracy of 40% yielding an F1-score of 39%.

The COVID-19 BERT and COVID-19 RoBERTa models which are fine-tuned on the COVID-19 dataset presented in [4], performed significantly better than the other pre-trained models i.e., the COVID-19 BERT model achieved an overall precision of 49%, an overall accuracy of 47% yielding an F1-score of 45%, while the COVID-19 RoBERTa model achieved a similar result with an overall precision of 48%, overall accuracy of 47% yielding an F1-score of 45%.

This is easily explained if one considers that the first set of models are largely based on the training of tweets from countries in the Global North with more general use-cases, while our models were trained on South African tweets with the specific use-case of vaccine hesitancy—in which the cultural and linguistic differences in the way people communicate in the two respective hemispheres clearly played a role in overall model performance. Moreover, there is a significant overlap or correlation between the emotional states of people when discussing M-pox, and the extent of displaying vaccine hesitancy. It is worth mentioning that in contrast to NLP-Town BERT and Cardiff-NLP RoBERTa, the accuracy of COVID-19 BERT and COVID-19 RoBERTa is superior to TextBlob automated labeling (Table 1). Considering that COVID-19 BERT and COVID-19 RoBERTa have previously been fine-tuned on COVID-19 dataset presented in [4], this superiority verifies that fine-tuning on manually labeled data boosts the accuracy and is advantageous to automated labeling.

Before fine-tuning the pre-trained models on the M-pox dataset, VADER had the maximum accuracy among all the models (Tables 1 and 3). However, after fine-tuning the pre-trained models on the M-pox dataset, their performances got remarkably better. The fine-tuned COVID-19 RoBERTa model outperformed other models and registered an overall precision of 70%, overall accuracy of 69% yielding an F1-score of 69%. Comparatively, even though the pre-trained models performed quite poorly when tested on the M-pox dataset (i.e. they all had an overall accuracy lower than 50%), they significantly outperformed the VADER and TextBlob models (Table 1), by up to 38% and 72.5% increase in the accuracy, respectively.

### Topic modelling

Topic modeling was performed on the misclassified tweets of the COVID-19 RoBERTa model before and after fine-tuning. The top 10 Most Frequent Terms per LDA Cluster for the two cases and their associated leading topics are shown in Table 4.

**Table 4:** Comparing LDA Results of the COVID-19 RoBERTa Mislabelled M-pox tweets before (top-section) and after (bottom-section) training. The M-pox dataset is from May 1st to Sep 5th, 2022.

Topic ID	Top 10 Salient words	Token Contribution	Inferred Topic
1	M-pox, vaccine, travel, supply, vaccination side, effect, response, safe, prevent	33.5%	Vaccine Safety and Availability Concerns
2	cases, M-pox, deadly, sores, blisters death, recovery, harmless, population, rate	22.9%	Fear of Death from M-pox Infection
3	Pox, monkey, transmit, airborne, mask water, hand, touch, animal, catch	20.3%	Possible Modes of M-pox Transmission
4	Covid, new, M-pox, mutation, lies, media, fake, mutation, corruption, greed	11.7%	Conspiracy Theories about the M-pox outbreak
5	Pox, covid, new, gay, sex, racist monkey, African, black, border	11.6%	M-pox related Stigmatization and Discrimination
Topic ID	Top 10 Salient words	Token Contribution	Inferred Topic
1	M-pox, virus, covid, spread, gay sex, condom, risk, apply, concern	27.6%	M-pox as a Sexually Transmitted Disease (STD)
2	M-pox, vaccine, side, effects, safe available, supply, smallpox, outbreak, death	23.4%	Vaccine Safety and Availability Concerns
3	M-pox, covid, hoax, mask, variant outbreak, variant, global, cause, want	19.5%	Conspiracy Theories about the M-pox Outbreak
4	Pox, monkey, get, real, scary sores, ugly, picture, cream, scratch	17.2%	Fear of M-pox related skin lesions and scarring
5	New, infection, pandemic, shutdown M-pox, kill, death, again, global, public	12.3%	Potential Emergence of a deadly M-pox pandemic

**Fig 2:**
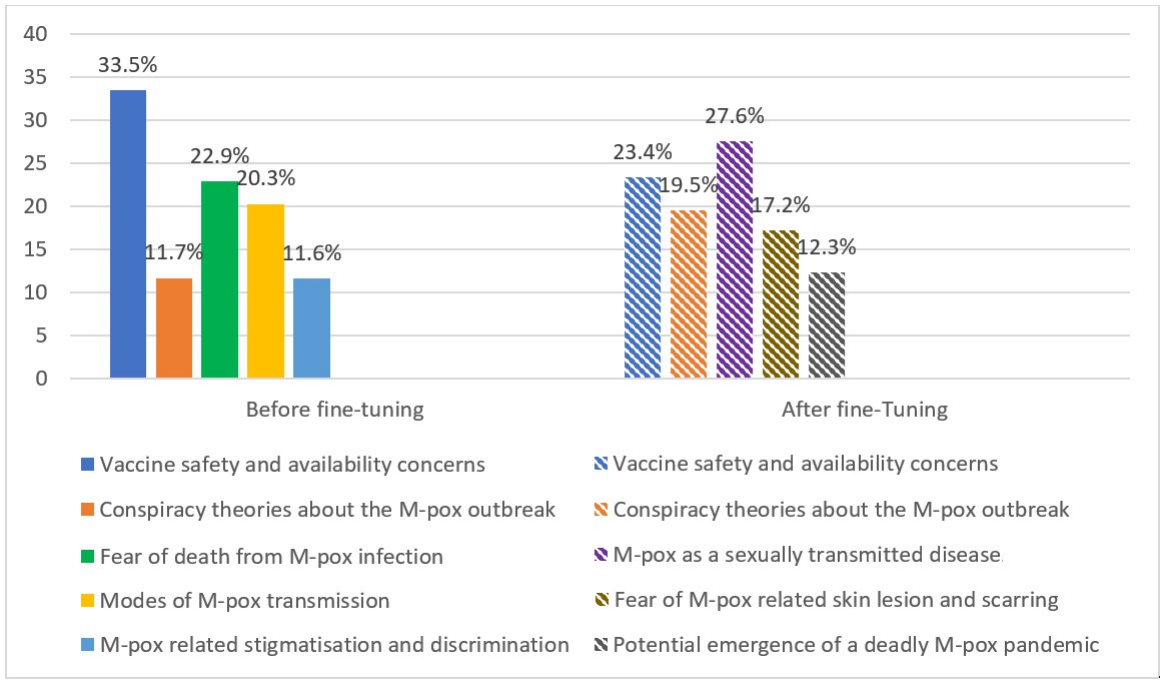
Distribution of the general topics in the sample space of M-pox tweets miss-classified by the RoBERTa model pre- and post-training.

From the results, one can see that the set of topics in both LDAs involve discussions or themes pertaining to vaccination, transmission, fear and panic, recovery, treatment as well as accompanying conspiracy theories. With respect to the LDA for the COVID-19 RoBERTa model prior to training, the topic with the highest contribution was “Vaccine Safety and Availability Concerns” at ≈ 34%, which also happened to be common to both LDA reports and also the second highest topic for the COVID-19 RoBERTa model post-training at ≈ 24%. The only other topic that was identical in both LDA analyses was given by: “Conspiracy Theories about the M-pox Outbreak,” which contributed ≈ 12% pre-training, but ≈ 20% post-training. The topic with the lowest contribution pre-training was taken by “M-pox related Discrimination and Stigmatization” at ≈ 12%, which correlates to the topic of lowest contribution for the case of the RoBERTa model post-training, since this discrimination and stigmatization is closely related or proportional to the degree of fear of M-pox being the next pandemic at ≈ 12%. It appears that after training the model topic contribution decreased for topics referring to problematic M-pox symptoms by ≈ 6%, topics pertaining to vaccination by ≈ 10%, but increased for topics pertaining to conspiracy theories by ≈ 8%. This shows that fine-tuning the M-pox dataset improved the model for classifying tweets discussing topics related to M-pox symptoms and vaccination more than tweets related to topics discussing conspiracy theories. Fig 2 displays the misclassified topics before and after fine-tuning the COVID-19 RoBERTa model, respectively.

Only two topics survived post-training i.e., “Vaccine Safety and Availability Concerns” (33.5% → 27.6%) and “Conspiracy Theories about the M-pox Outbreak” (11.7% → 19.5%). The topics that disappeared in leading order were: “Fear of Death from M-pox Infection” (22.9%), “Possible Modes of M-pox Transmission” (20.3%) and “M-pox-related Stigmatization and Discrimination” (11.6%). The emergent topics in leading order were: “M-pox as an STD” (27.6%), “Fear of M-pox related skin lesions and Scarring” (17.2%) and the “Potential Emergence of a Deadly M-pox Pandemic” (12.3%). It is apparent that although 3 out of the 5 topics in the one set of LDA results are not identical to the other; they are in essence the same entity under some grouping i.e. topics within a general topic. For example, “Possible Modes of M-pox Transmission” and “M-pox as an STD.” are sub-topics of the general topic of M-pox transmission and infectivity. We also found that “Fear of M-pox related skin lesions and scarring” and “Fear of death by M-pox” are sub-topics of M-pox infection and recovery; “Potential Emergence of an M-pox pandemic” and “M-pox related Stigmatization and Discrimination,” are sub-topics of public mass panic and hysteria. The COVID-19 RoBERTa model improved after fine-tuning and shifted the misclassified topics to specific topics. This leads to the conclusion that existing pre-trained models better be fine-tuned on hand-labeled datasets in order to perform well for specific purposes. Moreover, by comparing Tables 1 and 2, we observe that models that are fine-tuned on hand-labeled data have higher accuracy compared to common models used for NLP, such as VADER and TextBlob.

## Discussion

In this work, we evaluated lexicon-based automated labeling for stance analysis of a Twitter dataset related to M-pox. We borrowed two models from [4], named, COVID-19 BERT and COVID-19 RoBERTa, which were fine-tuned on a dataset related to COVID-19 vaccines manually labeled for stance detection [4]. Table 5 summarizes the results, in the order of the model accuracy.

**Table 5:** Summary of the results.

Model	Precision	Recall	F1-score	Accuracy
NLP-Town BERT	43	39	40	39
Cardiff-NLP RoBERTa	40	40	39	40
TextBlob Automated Labels	41	40	37	40
COVID-19 BERT	49	47	45	47
COVID-19 RoBERTa	48	47	45	47
VADER Automated Labels	49	50	47	50
Fine-Tuned NLP-Town	60	61	60	60
Fine-Tuned Cardiff-NLP	58	62	60	59
Fine-Tuned BERT	69	68	68	68
Fine-Tuned RoBERTa	70	69	69	69

We compared the accuracy of VADER and TextBlob with COVID-19 BERT and COVID-19 RoBERTa, as well as NLP-Town BERT and Cardiff-NLP RoBERTa. VADER and TextBlob had an accuracy higher than NLP-Town BERT and Cardiff-NLP RoBERTa; however, the accuracy of COVID-19 BERT and COVID-19 RoBERTa was higher than TextBlob. This shows the efficacy of fine-tuning transformer-based models on stance detection hand-labeled data, even if the data is from a different, but relatively close context. Nevertheless, all the lexicon-based and transformer-based models performed poorly on the M-pox dataset, with an accuracy smaller or equal to 50%.

Other papers that have evaluated the accuracy of VADER and TextBlob for automated labeling, report slightly higher accuracies, however they have evaluated the lexicon-based models for sentiment analysis [21–23]. Stance-detection is a more complicated task that requires sensitive operations, such as describing an annotation outline, defining annotation guidelines, and training annotating experts for building a consensus dictionary [24]. Automated stance detection models could have incredibly low accuracies; therefore, they should be avoided for supervised machine learning, especially for sensitive health-related tasks such as vaccine hesitancy identification, pandemic modeling, disease detection, and public opinion tracking.

Next, we fine-tuned the transformer-based models on the manually labeled M-pox dataset, which boosted their accuracies to higher than lexicon-based automated labels. We conclude that automated labels do not reach the accuracy of models fine-tuned on manually labeled stance detection datasets and are significantly far from them. Similar to other papers [35], the COVID-19 RoBERTa model provided the highest accuracy after being fine-tuned; therefore, to study the effect of fine-tuning on manually labeled data further, we used LDA to perform topic analysis on the misclassified tweets, before and after fine-tuning COVID-19 RoBERTa model. The results indicated that after fine-tuning the scope of misclassified tweets became smaller and a subset of the scope of pre-fine-tuning misclassified tweets. This shows the efficacy of fine-tuning on manually labeled data for building an accurate stance-detection model.

## Conclusion

Social media platforms are powerful tools that could help policy-makers and health authorities control and contain epidemics and manage emergencies. Social outcomes of emergent crises such as M-pox and COVID-19 pandemic, e.g. stigmatization, vaccine hesitancy, resistance against PIs and NPIs, could be alleviated only with social impositions and dissemination of true information [15, 16, 19]. Therefore, researchers have focused on developing NLP models that could extract meaningful information from mass opinions and social media conversation to inform decision-making. This study has shed light on the effectiveness of lexicon-based models for stance detection in the context of the M-pox outbreak. However, several limitations and implications for future research and policy should be considered.

One of the main limitations of this study is the reliance on a manually labeled dataset, which can be labor-intensive and may introduce bias. Future research could explore more efficient labeling methods, such as semi-supervised learning or active learning, to improve the scalability and generalizability of stance detection models.

Another limitation is the focus on Twitter data, which may not fully represent the diversity of opinions and attitudes expressed in other social media platforms or offline contexts. Future studies could explore the use of multi-modal data sources to capture a more comprehensive view of public opinion during health crises.

From a practical standpoint, our findings suggest that policymakers and healthcare authorities should be aware of the diverse opinions and attitudes expressed on social media during health crises. By understanding these perspectives, authorities can tailor their communication strategies to address public concerns and mitigate misinformation.

Overall, this study highlights the importance of leveraging advanced machine learning techniques for stance detection in health-related contexts. By addressing the limitations and implications discussed, future research can contribute to more effective crisis management and public health policies in the face of emerging epidemics and pandemics.

## Supporting information

S1 File This file includes the ID of the tweets and their stance labels which are from three classes namely, positive, neutral, and negative. (CSV)
